# CD117^+^ Dendritic and Mast Cells Are Dependent on RasGRP4 to Function as Accessory Cells for Optimal Natural Killer Cell-Mediated Responses to Lipopolysaccharide

**DOI:** 10.1371/journal.pone.0151638

**Published:** 2016-03-16

**Authors:** Saijun Zhou, Kumiko Tanaka, Meredith O’Keeffe, Miao Qi, Fatima El-Assaad, James C. Weaver, Gang Chen, Christopher Weatherall, Ying Wang, Bill Giannakopoulos, Liming Chen, DeMint Yu, Matthew J. Hamilton, Lislaine A. Wensing, Richard L. Stevens, Steven A. Krilis

**Affiliations:** 1 Department of Infectious Diseases, Immunology, and Sexual Health, St. George Hospital, and the St. George and Sutherland Clinical School, Faculty of Medicine, University of New South Wales, Sydney, New South Wales, Australia; 2 Laboratory of Hormones and Development (Ministry of Health), Metabolic Hospital and Tianjin Institute of Endocrinology, Tianjin Medical University, Tianjin, TJ, China; 3 Dendritic Cell Research Laboratory, Immunity Vaccines and Immunisation, Burnet Institute, Prahran, Melbourne, Victoria, Australia; 4 Department of Cardiology, St. George Hospital, Sydney, New South Wales, Australia; 5 School of Biomedical Sciences and Pharmacy, University of Newcastle, Callaghan, New South Wales, Australia; 6 Division of Gastroenterology, Brigham and Women’s Hospital, Boston, MA, United States of America; 7 Departament of Immunology, Biomedical Sciences Institute, University of São Paulo, São Paulo, Brazil; Johns Hopkins University School of Medicine, UNITED STATES

## Abstract

Ras guanine nucleotide-releasing protein-4 (RasGRP4) is an evolutionarily conserved calcium-regulated, guanine nucleotide exchange factor and diacylglycerol/phorbol ester receptor. While an important intracellular signaling protein for CD117^+^ mast cells (MCs), its roles in other immune cells is less clear. In this study, we identified a subset of *in vivo*-differentiated splenic CD117^+^ dendritic cells (DCs) in wild-type (WT) C57BL/6 mice that unexpectedly contained RasGRP4 mRNA and protein. In regard to the biologic significance of these data to innate immunity, LPS-treated splenic CD117^+^ DCs from WT mice induced natural killer (NK) cells to produce much more interferon-γ (IFN-γ) than comparable DCs from RasGRP4-null mice. The ability of LPS-responsive MCs to cause NK cells to increase their expression of IFN-γ was also dependent on this intracellular signaling protein. The discovery that RasGRP4 is required for CD117^+^ MCs and DCs to optimally induce acute NK cell-dependent immune responses to LPS helps explain why this signaling protein has been conserved in evolution.

## Introduction

The four members of the Ras guanine nucleotide-releasing protein (RasGRP) family of intracellular signaling proteins in mice and humans activate small GTPases that participate in varied cellular processes [1,2]. RasGRP4 was initially cloned in a hunt for novel transcripts expressed by interleukin (IL)-3-dependent mouse bone marrow-derived mast cells (mBMMCs) [3]. A homology-based screening approach was then used to isolate its rat and human orthologs. All examined populations of mature CD117^+^ MCs contain RasGRP4 mRNA and protein [3–5].

Although RasGRP4 is not essential for the development of granulated MCs in mice, this signaling protein participates in the release of some of the cell’s pro-inflammatory mediators [6–9]. RasGRP4 is more restricted than RasGRP1, RasGRP2, or RasGRP3. Of the ~9 million expressed sequence tags (ESTs) in the human database, only 14 RasGRP4 ESTs have been isolated from normal human tissue. While most of these were from the bone marrow where MC-committed progenitors reside, two were from the spleen. Because MCs are rarely found in the spleens of normal mice and humans, the latter EST data raised the possibility that another immune cell that constitutively resides in the spleen expresses this signaling protein.

Using a homologous recombination approach, we recently created a RasGRP4-null C57BL/6 (B6) mouse line [9]. We then showed that experimental arthritis and colitis were markedly reduced in these transgenic animals relative to that of wild-type (WT) B6 mice. Despite the adverse roles of RasGRP4 in two inflammatory disease mouse models, the fact that humans have a functional RasGRP4 gene implies that this signaling protein has been conserved for >85 million years because of its importance to our survival in some way.

CD117/KIT [10,11] is a tyrosine-kinase receptor expressed on the outer surfaces of hematopoietic stem cells. It recognizes the membrane-bound cytokine KIT ligand/stem cell factor [12,13]. In most mature hematopoietic cells, the expression of CD117 is lost during the final stages of differentiation. Nevertheless, some populations of dendritic cells (DCs) retain their expression of CD117 throughout their development [14,15], analogous to mature MCs [16]. The latter granulocytes [17] and DCs [18,19] can function as accessory cells in inflammation and innate immune responses, in part, by inducing natural killer (NK) cells to acutely increase their expression of interferon-γ (IFN-γ) and other pro-inflammatory cytokines. The expression and release of IFN-γ by DC-stimulated NK cells is important in combating pathogens while adaptive immune responses are being primed, and CD117 is important for these responses [20].

The presence of infectious agents can be sensed by DCs which, in turn, activate NK cells in contact-dependent and -independent manners. MCs also can sense pathogens via their complement and Toll-like receptors (TLRs) [21,22], and some of the cell’s exocytosed mediators have beneficial roles in infections (for review [23]). Despite these data, the factors and mechanisms that are responsible for optimal DC- and MC-NK cell crosstalk remain to be elucidated at the molecular level. We now demonstrate beneficial roles for RasGRP4 in different arms of innate immunity triggered by lipopolysaccharide (LPS)-TLR-signaling pathways.

## Materials and Methods

### Mice

WT B6 mice were obtained from the Animal Resources Centre (Perth, Australia). The creation of the RasGRP4-null B6 mouse line by us has been previously described [24]. These transgenic mice were initially housed in the Australian BioResources Facility in Moss Vale, Australia. However, both populations of B6 mice were housed in the Animal Facility at the University of New South Wales at least one wk before experimentation. All animal studies were approved by the Animal Ethics Committee of the University of New South Wales, Sydney, Australia. As mandated by our Institution, the method of euthanasia of the mice used in this study consisted of anesthesia (5% isoflurane) followed by cervical dislocation.

### Isolation of NK cells, CD117^-^ cells, and CD117^+^ cells from the mouse’s spleen

Splenocytes from 9–12 wk-old male WT and RasGRP4-null B6 mice were isolated by teasing dispersed spleens through a nylon mesh with 70-μm pores (BD Biosciences, San Jose, CA) in RPMI-1640 medium containing 10% fetal calf serum, 100 U/mL penicillin, 100 μg/mL streptomycin, and 2 mM L-glutamine. In each instance, the resulting single-cell suspension was washed twice with the above medium and the total number of cells was determined using a hemocytometer.

A magnetic-bead antibody (Ab) separation approach using StemCell Technologies’ EasySep kit (Vancouver, Canada) was employed to purify NK cells from the dispersed mouse spleen by negative selection [25]. Cell suspensions of splenocytes were washed twice and incubated with a cocktail of biotinylated Abs that recognize non-NK cells. This was followed by the EasySep® biotin selection procedure. The resulting cells were suspended in enriched RPMI medium at a density of 5 x 10^6^ cells/mL. Their purity was assessed by flow cytometry using anti-NK1.1 and anti-CD3 Abs.

Splenocytes from naïve WT and RasGRP4-null B6 mice also were segregated based on their surface expression of CD117. To obtain these two populations of cells in each mouse strain, 1 x 10^7^ splenocytes were suspended in 1 mL of phosphate buffered saline (PBS) containing 2 mM EDTA and 2% fetal calf serum. The cells suspension were incubated with rat anti-mouse CD16/CD32 Ab (BD Biosciences) to block the non-specific binding of immunoglobulins to Fcγ receptors. Following the addition of a biotinylated rat anti-mouse CD117 Ab (BD Biosciences), each cell suspension was incubated for 15 min at 4˚C. The treated cells were washed twice, and then suspended in 70 μL of the above buffer containing magnetic microbeads conjugated to monoclonal anti-biotin antibody (Miltenyi Biotec, Bergisch Gladbach, Germany).

After a 15-min incubation at 4˚C, the cell suspensions were washed twice with buffer and then loaded onto a MACS® column, which was placed in the magnetic field of a MACS separator (Miltenyi Biotec). The CD117^-^ non-labeled splenocytes that failed to bind to the column were washed and suspended in serum-enriched RPMI-1640 medium at a cell density of 5 x 10^6^ cells/mL. The CD117^+^ splenocytes that bound to the column were liberated by switching off the magnetic field, followed by washing the column and the liberated cells with medium. The CD117^+^ and CD117^-^ splenocytes were also magnetically separated using the EasySep™ mouse CD117^+^ selection kit (Miltenyi Biotec) that employs a phycoerythrin-labeling reagent that recognizes the tyrosine kinase receptor on the surfaces of cells.

Monoclonal Abs were used that recognize numerous proteins on the surfaces of the CD117^-^ and CD117^+^ splenocytes obtained from WT and RasGRP4-null B6 mice. Single cell suspensions were initially exposed to a rat anti-mouse CD16/CD32 Ab (BD Biosciences) to block non-specific antibody binding to Fcγ receptors. A panel of fluorochrome-conjugated Abs (S1 Table) was then used in varied combinations to phenotype the different populations of immune cells in the spleens of WT and RasGRP4-null mice. Surface marker staining was performed using a modification of a previously described method for the staining of mononuclear cells [26]. Briefly, enriched CD117^+^ and CD117^-^ splenocytes were incubated for 30 min in the dark on ice in 20 μL of PBS containing 0.5% bovine serum albumin, 0.1% sodium azide), and 2 mM EDTA in the presence of different combinations of Abs that recognize CD1d, CD3, CD4, CD8, CD11b, CD11c, CD14, CD21/CD35, CD25, CD45R, CD117, NK1.1, and Gr-1 (BD Biosciences and Miltenyi Biotec).

### Isolation of splenic DCs and evaluation of their expression of RasGRP4

DCs were isolated from the spleens of WT and RasGRP4-null mice employing a widely used procedure [27,28]. Briefly, spleens were digested with DNase and collagenase, and the resulting cells were collected after centrifugation in 1.08 g/mL Nycodenz to remove contaminating granulocytes which were of higher density than DCs. The resulting cells were then incubated with a depletion cocktail containing a combination of rat mAbs that recognize mouse CD19, CD3, Thy1.1, TER-119, and Ly6G. The non-DC populations of cells were removed using anti-rat IgG magnetic beads (Qiagen Inc, Valencia, CA). DCs also were magnetically isolated using the EasySep™ mouse pan-DC enrichment kit that contains biotinylated antibodies against cell surface antigens expressed on non-DCs.

For RasGRP4 immunostaining, a rabbit anti-mouse RasGRP4 Ab we previously generated against a novel 12-mer peptide sequence in the signaling protein [5] was used with the BD Intrasure kit (BD Biosciences, Franklin Lakes, NJ), according to the manufacturer's instructions. After the cells were exposed to the relevant Abs, they were fixed with Reagent A, followed by BD FACS lysing solution (BD Biosciences). They were permeabilized with Reagent B and stained with the rabbit anti-mouse RasGRP4 Ab [5] for 30 min, followed by Alexa488-labeled anti-rabbit IgG (Life Technologies, Carlsbad, CA). The characteristics of the other conjugated Abs used in the flow cytometry analyses are listed in the S1 Table. In each of these experiments, ~20,000 cells per sample were analyzed using a FACS Calibur flow cytometer (BD Biosciences). The obtained data were analyzed using FlowJo software (Tree Star Inc, Ashland, OR).

### Expression of cytokines and chemokines by LPS-activated NK cells, CD117+ splenocytes, and the co-cultured cells

Initially, 5 x 10^6^ unfractionated splenocytes were cultured at 37°C and 5% CO_2_ in 1 mL of RPMI medium in the absence or presence of 1 μg/mL of LPS (*E*. *coli* strain O111:B4; Sigma-Aldrich, St. Louis, MO) for 24 or 48 h. Purified NK cells were also co-cultured with CD117^+^ splenocytes, each at 5.0 x 10^5^ cells in 100 μL of RPMI medium. The cells were mixed in U-bottom 96-well plates (Greiner Bio-One, Frickenhausen, Germany), and then incubated in the presence of 1 μg/mL of LPS (Sigma-Aldrich).

Transwell experiments were next performed using 48-well plates containing 0.4-μm pore cut-off membranes (Corning, New York) to prevent physical contact between different populations of cells. For these experiments, purified NK cells and purified CD117^+^ splenocytes from naïve B6 mice were cultured at ratios of 1:1 and 1:2 for 24 and 48 h, respectively, in the absence or presence of 1 μg/mL LPS. The latter cells from WT mice were placed in the lower chamber, whereas the purified NK cells from WT mice were placed in the upper chamber. Following this step, the cells and supernatants were separated by centrifugation (2000 x g for 5 min). The supernatants were stored at -80°C until the levels of cytokines and chemokines could be measured.

The levels of IL-2, IL-4, IL-6, IL-10, IL-12p70, IL-17A, IFN-γ, chemokine (C-C motif) ligand 2 (CCL2), and tumor necrosis factor (TNF)/TNFSF4 were determined using BD Biosciences’ cytometric bead array kit for mouse Th1/Th2/Th17 cytokines and chemokines and the mouse inflammation kit for inflammation cytokines, according to the manufacturer’s instructions. Standard curves were determined for each cytokine and chemokine from 20 to 5,000 pg/mL. The acquired data were analyzed with the FCAP Array software (Soft Flow Hungary, Kedves, Hungary) by applying the four-parameter-curve fit option. The minimum detection levels for IL-2, IL-4, IL-6, IFN-γ, TNF/TNFSF4, IL-17A, and IL-10 in the mouse Th1/Th2/Th17 cytokine kit were 0.1, 0.03, 1.4, 0.5, 0.9, 0.8, and 16.8 pg/mL, respectively. The minimum detection levels for IL-6, IL-10, CCL2, IFN-γ, TNF/TNFSF4, and IL-12p70 in the mouse inflammation kit were 5.0, 17.5, 52.7, 2.5, 7.3, and 10.7 pg/mL, respectively. The Bio-plex Pro^TM^ mouse cytokine GrpI panel 23, the Bio-plex Pro^TM^ mouse cytokine GrpII panel 9, and the Bio-Plex 200 System (Bio-Rad Laboratories, Hercules, CA) were used to detect those cytokines which were expressed when B6 mouse CD117^+^ splenocytes were exposed to LPS. The detection concentration range of these two kits was 0.2–32,000 pg/mL.

After 24 h of stimulation with 1 μg/mL of LPS, the treated DC-rich CD117^+^ splenocytes from WT and RasGRP4-null B6 mice were evaluated for the presence of IFN-γ using a Miltenyi Biotec cytokine detection kit. For the latter assay, the cells were incubated for 10 min with an IFN-γ-specific Ab conjugated to allophycocyanin. To identify the primary IFN-γ-expressing cells in the splenocyte preparations, the LPS-treated cells were stained with anti-CD3, anti-CD11b, anti-CD45R, anti-NK1.1, or anti-Gr-1 Abs, followed by propidium iodide or 7-aminoactinomycin D. They were washed twice with PBS containing 0.5% bovine serum albumin, 0.1% azide, and 2 mM EDTA, and then analyzed on a FACS Canto II flow cytometer.

### Generation of mBMMCs and co-culture of these non-transformed MCs with mouse NK cells and CD117^-^ splenocytes

Bone marrow cells from the femurs and tibias of WT and RasGRP4-null B6 mice were cultured at 37°C in a 5% CO_2_ incubator at a starting density of 5 x 10^5^ cells/mL in RPMI-1640 medium (Invitrogen, Carlsbad, CA) supplemented with 10% heat inactivated fetal calf serum (Invitrogen), 100 U/mL penicillin, 100 μg/mL streptomycin, 2 mM L-glutamine, 0.1 mM non-essential amino acids, 50 μM 2-mercaptoethanol, and 10 ng/mL recombinant mouse IL-3 (R&D Systems, Minneapolis, MN), as previously described [29]. The culture medium was changed every 3 d. After 4 wk of culture, we and others previously showed that >98% of the cells in the resulting cultures were MCs as assessed by their surface expression of FcεRI, IL-33, and CD117, and by the presence of histamine, serglycin proteoglycans, mouse MC protease (mMCP)-5, mMCP-6, and MC carboxypeptidase A3 in their secretory granules [29–33].

Different combinations of mBMMCs, mouse CD117^-^ splenocytes, and enriched mouse NK cells (each at 2.5 x 10^5^ cells in 50 μL of RPMI medium) were placed in U-bottom 96-well plates (Greiner Bio-One, Frickenhausen, Germany) and then incubated in the absence or presence of 1 μg/mL of LPS (Sigma-Aldrich). Following this step in each experiment, the cells and supernatants were separated by centrifugation. The supernatants were stored at -80°C until their cytokine and chemokine levels could be measured as described above. In contrast, the LPS-treated cells were immediately subjected to flow cytometry.

### Transcript analyses of DC-rich CD117^+^ splenocytes from WT and RasGRP4-null mice before and after exposure to LPS

DC-rich CD117^+^ splenocytes were isolated from male WT (n = 5) and RasGRP4-null (n = 5) B6 mice, as indicated above. Five million CD117^+^ splenocytes/mL were challenged with 1 μg/mL of LPS for varying times (0, 12, 24, 36, and 48 h). At each time point, the treated cells were processed for RNA extraction with the RNeasy® Plus Mini kit (Qiagen Inc), according to the manufacturer's instructions. Total RNA was isolated and its purity in each instance was determined. An aliquot of each sample was subjected to gel electrophoresis to confirm the presence of intact 18 and 28S rRNA.

The ‘One-Cycle Eukaryotic Target Labeling Assay Protocol’ recommended by Affymetrix was used by the Clive and Vera Ramaciotti Centre (University of New South Wales, Sydney, Australia) to generate the biotin-labeled cRNAs that were hybridized to Affymetrix MoGene-2.0-st Exon/Gene arrays (Affymetrix, Santa Clara, CA). Before the microarray analyses, the amount and quality of the generated cRNAs were determined on an Agilent 2100 Bioanalyzer (Agilent Technologies, Inc., Santa Clara, CA).

Ten GeneChips were used in these microarray experiments (namely DC-rich CD117^+^ splenocytes from WT and RasGRP4-null mice cultured in the absence or presence of LPS for 12, 24, 36, and 48 h). The resulting data were analyzed using Affymetrix® Expression Console™ Software (Affymetrix), as well as by a more stringent multi-step approach [34]. In the latter method, the microarray data from the 10 samples were first normalized to that of the transcript that encodes glyceraldehyde 3-phosphate dehydrogenase (GAPDH). To minimize false positives and negatives, we decided that the level of a transcript in a LPS-treated splenocyte sample had to be >1% that of the latter housekeeping transcript to be considered present. Its level then had to be >3.6 higher (or lower) than that in the untreated splenocytes. The latter arbitrary cut-off value was chosen based on a maximal 3.6-fold difference in the levels of the CD117 transcript in the ten samples (see S2 Table).

### Blocking Experiment

Enriched NK cells and DC-rich CD117^+^ splenocytes from WT mice were cultured in a 96-well plate at a density of 5 x 10^6^ cells/mL in the ratio of 1:1 in the presence of 20 μg/mL of a rat Ab directed against TNFSF4 (eBioscience). Supernatants were collected by centrifugation at 250 x g for 10 min and the levels of IFN-γ were measured as described above.

### LPS treatment of WT and RasGRP4^-/-^ mice, and detection of intracellular IFN-γ in their peripheral blood mononuclear cells and splenocytes

Age-matched male WT and RasGRP4-null B6 mice were given by intravenous injection pyrogen-free saline lacking or containing LPS (*E*.*coli* LPS, serotype 0111:B4) Sigma-Aldrich) at 2 μg/g mouse body weight. One hundred and fifty min later, the treated mice were euthanized, and approximately 1 mL of whole blood was collected by cardiac puncture with a syringe containing 10 μL of 250 mM EDTA. The levels of IFN-γ, IL-6, IL-10, IL-12p70, TNF/TNFSF4, and CCL2 in each animal’s plasma were measured.

The cells in the peripheral blood of each mouse were also obtained for cytokine and chemokine analyses. For these experiments, ~1 mL of whole mouse blood was collected immediately after euthanasia by cardiac puncture with a syringe containing 10 μL of 250 mM EDTA. Each blood sample was centrifuged at 200 x g for 15 min. The resulting cell pellets were exposed to erythrocyte-lysis buffer (Sigma-Aldrich) for 2 min to remove contaminating erythrocytes. Each cell suspension was then washed twice with PBS. Total cell numbers were determined using a hemocytometer. The resulting peripheral blood cells and splenocytes were evaluated for IFN- γ expression using a Miltenyi Biotec’s mouse cytokine detection kit (Miltenyi Biotec). To identify the primary IFN-γ-expressing cells in the peripheral blood and spleen, the IFN-γ^+^ cells were stained with anti-CD3, anti-CD11b, anti-CD45R, anti-NK1.1, anti-CD4, and anti-CD8 Abs, followed by 7-aminoactinomycin D.

### Statistical analysis

The relevant parametric data sets were compared by paired two-tailed Student's t test using Graph Pad Prism 5 software (Graph Pad, San Diego, CA). Probability (p) values of < 0.05 were considered statistically significant.

## Results

### Evaluation of the percentages of B cells, DCs, T cells, and NK cells in the peripheral blood, spleen, and/or thymus of WT and RasGRP4-null B6 mice

Although all examined mouse and human MCs expressed RasGRP4 [3,5], we previously showed that there was no decrease in the numbers of mature MCs in the skin and peritoneal cavities of our RasGRP4-null B6 mice, as well as their committed progenitors in the bone marrow [9]. Nevertheless, RasGRP1 is essential for the development of mature T cells [35], and both RasGRP1 and RasGRP3 participate in B cell development and proliferation [36]. In order to determine whether or not RasGRP4 is essential for the development of B cells, DCs, T cells, and/or NK cells, we quantitated the numbers of these hematopoietic cells in the peripheral blood, spleen, and/or thymus of WT and RasGRP4-null B6 mice. There was no significant difference in the weights of the spleen [60.3 ± 11.5 mg in WT mice versus 74.0 ± 22.6 mg in RasGRP4-null mice (mean ± SD, n = 5, p = 0.51)] or thymus [57.4 ± 6.8 mg in WT mice versus 72.2 ± 24.3 mg in RasGRP4-null mice (mean ± SD, n = 4, p = 0.57)].

**Fig 1:**
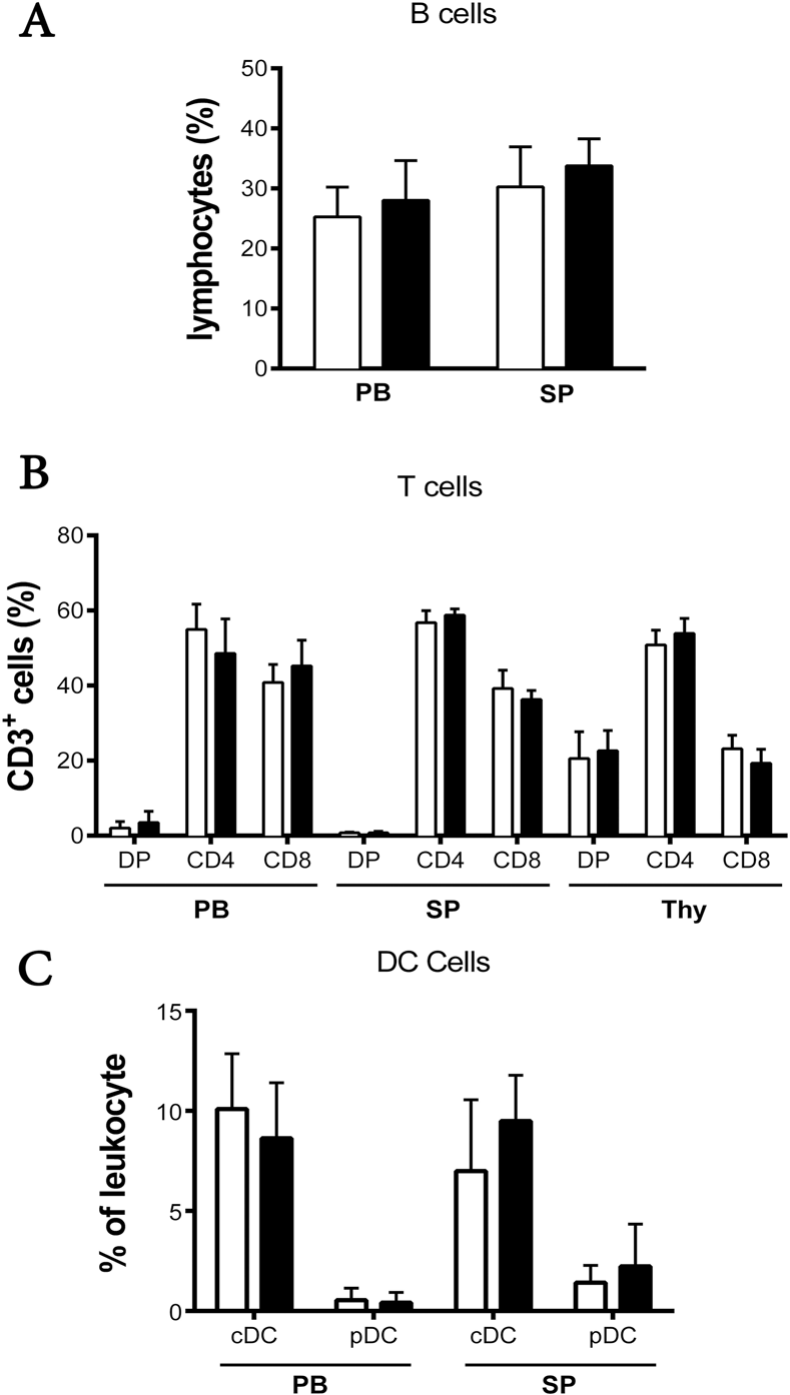
Percentages of B cells, T cells, and DCs in the peripheral blood, spleen, and thymus of RasGRP4-null and WT B6 mice. Flow cytometry was performed on the peripheral blood cells, splenocytes, and thymocytes from RasGRP4-null and WT B6 mice using specific Abs. The numbers represent the percentage of gated cells staining positive for their appropriate surface markers. (A) The B cells in the peripheral blood and spleen were gated from the viable lymphocyte gate from forward scattered light (FSC) vs. side scattered light (SSC). Most of these B cells had a B220^+^/CD23^+^/CD21^+^ phenotype. (B) T cells were identified from the lymphocyte gate based on their CD3^+^/CD4^+^, CD3^+^/CD8^+^, or CD3^+^/CD4^+^/CD8^+^ surface phenotypes. (C) cDCs and pDCs were defined as CD11c^+^/CD45R^low^ and CD11c^+^/CD45R^high^, respectively. Data are the mean ± SD, n = 6 of WT (□) and RasGRP4-null (■) mice per group, in at least 4 experiments. * = p< 0.05. PB, peripheral blood; SP, spleen; DP, double positive; and Thy, thymus.

Flow cytometry was performed on the cells from the peripheral blood, spleen, and thymus of WT and RasGRP4-null B6 mice using Abs (S1 Table) that recognize B cells, DCs, T cells, and NK cells. The percentages of B cells, conventional DCs (cDCs), and plasmacytoid DCs (pDCs) in the peripheral blood and spleens of the two mouse strains were similar (n = 6) (Fig 1). Likewise, no significant differences were found in the percentages of the CD3^+^/CD4^+^ or CD3^+^/CD8^+^ mature T cells in the peripheral blood, spleen, or thymus of the two mouse strains. Finally, there was no difference in the numbers of splenic NK1.1^+^ cells in WT and RasGRP4-null mice (data not shown). Targeted inactivation of the RasGRP4 gene therefore did not lead to noticeable deficiencies in the number and distribution of B cells, DCs, T cells, and NK cells in the transgenic B6 mouse line.

### Attenuated LPS-mediated expression of IFN-γ in the unfractionated splenocytes of RasGRP4-null mice

RasGRP4 participates in the expression and release of cytokines from mBMMCs exposed to phorbol 12-myristate 13-acetate [9]. RasGRP4 ESTs have been isolated from normal human spleen (see UniGene Hs.130434), and RasGRP3 participates in TLR signaling pathways in macrophages [37]. Moreover, MCs express LPS-responsive TLRs [21,22]. Thus, to determine whether or not RasGRP4 plays a significant role in the expression of cytokines following LPS engagement of the TLRs on any of the cell types in the spleen, we assayed for a panel of cytokines following LPS stimulation of unfractionated splenocytes from WT and RasGRP4-null B6 mice.

**Fig 2:**
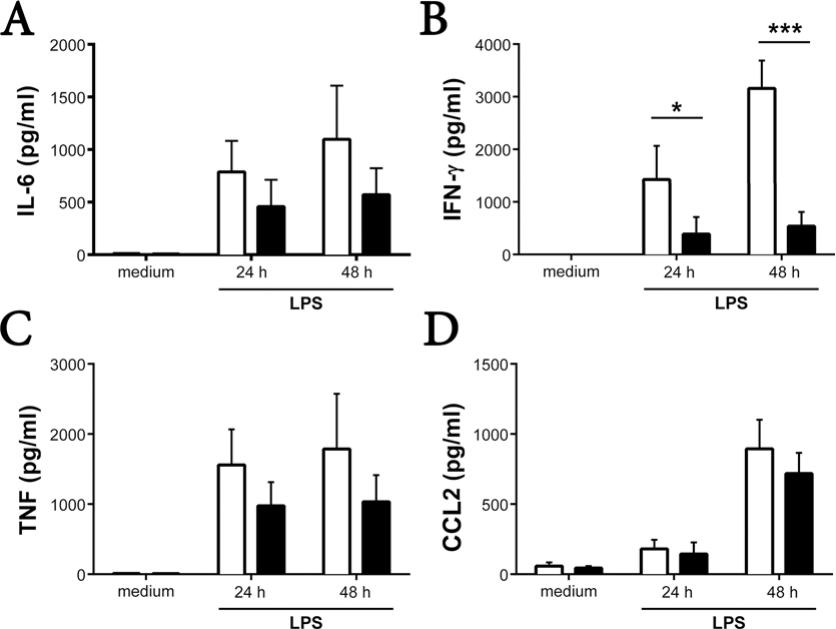
Pro-inflammatory cytokine production by unfractionated splenocytes in response to LPS. Unfractionated splenocytes from WT (□) and RasGRP4-null (■) mice were incubated for 24 or 48 h in the presence of 1 μg/mL of LPS. (A-D) The levels of IL-6 (A), IFN-γ (B), TNF/TNFSF4 (C), and CCL2 (D) were determined in the resulting supernatants. For negative controls, splenocytes from the two groups of mice were cultured 48 h in medium lacking LPS. The bar graphs represent the mean data ± SD, n = 4 (A-C) and n = 3 (D). * = p < 0.05, *** = p < 0.0005.

As anticipated, the bacterial product induced the unfractionated splenocytes from WT mice to generate large amounts of IFN-γ, IL-6, CCL2, and TNF/TNFSF4 relative to the unfractionated splenocytes from WT mice that did not encounter LPS (Fig 2A–2D). Although the levels of IL-6, TNF/TNFSF4, and CCL2 were modestly reduced in the splenocytes of the corresponding RasGRP4-null mice that encountered LPS for 24 or 48 h, the data were not statistically different from that of the treated WT mouse splenocytes. In contrast, there was a marked difference in the levels of IFN-γ produced by the unfractionated splenocytes from RasGRP4-null mice that encountered LPS for 48 h relative to similarly treated splenocytes from WT mice (540 ± 270 pg/mL versus 3160 ± 530 pg/mL, mean ± SD, n = 4, p = <0.005) (Fig 2B). In support of these protein data, the CD117^+^ splenocytes from WT mice markedly increased their levels of the IFN-γ transcript 24 h after they encountered LPS (S2 and S3 Tables; the raw microarray data also can be found at the Gene Expression Omnibus database at accession number GSE69011). This did not occur in the LPS-treated CD117^+^ splenocytes from RasGRP4-null mice (Fig 2B). Interestingly, the levels of the RasGRP4 transcript in the DC-rich CD117^+^ splenocytes from WT B6 mice decreased markedly 12–48 h after these cells encountered LPS (S2 Table).

IL-10 inhibits the production of IFN-γ when the splenocytes from WT mice are exposed to LPS, whereas IL-12 promotes the production of this anti-viral cytokine [38]. The levels of IL-12 protein (as well as the levels of IL-2, IL-4, and IL-17A) were below detection in the supernatants of LPS-stimulated splenocytes from both populations of B6 mice. Moreover, the LPS-treated splenocytes from these mice produced similar levels of IL-10, whether examined at the 24- or 48-h time points (data not shown). The RasGRP4-dependent expression of IFN-γ in the LPS-treated splenocytes from the two B6 mouse strains therefore did not appear to be a secondary consequence of altered expression of IL-2, IL-4, IL-10, IL-12, IL-17A, or CCL2.

### NK cells from WT mice express higher levels of IFN-γ after LPS-stimulation of undefined accessory cells in the spleens of WT mice but not RasGRP4-null mice

**Fig 3:**
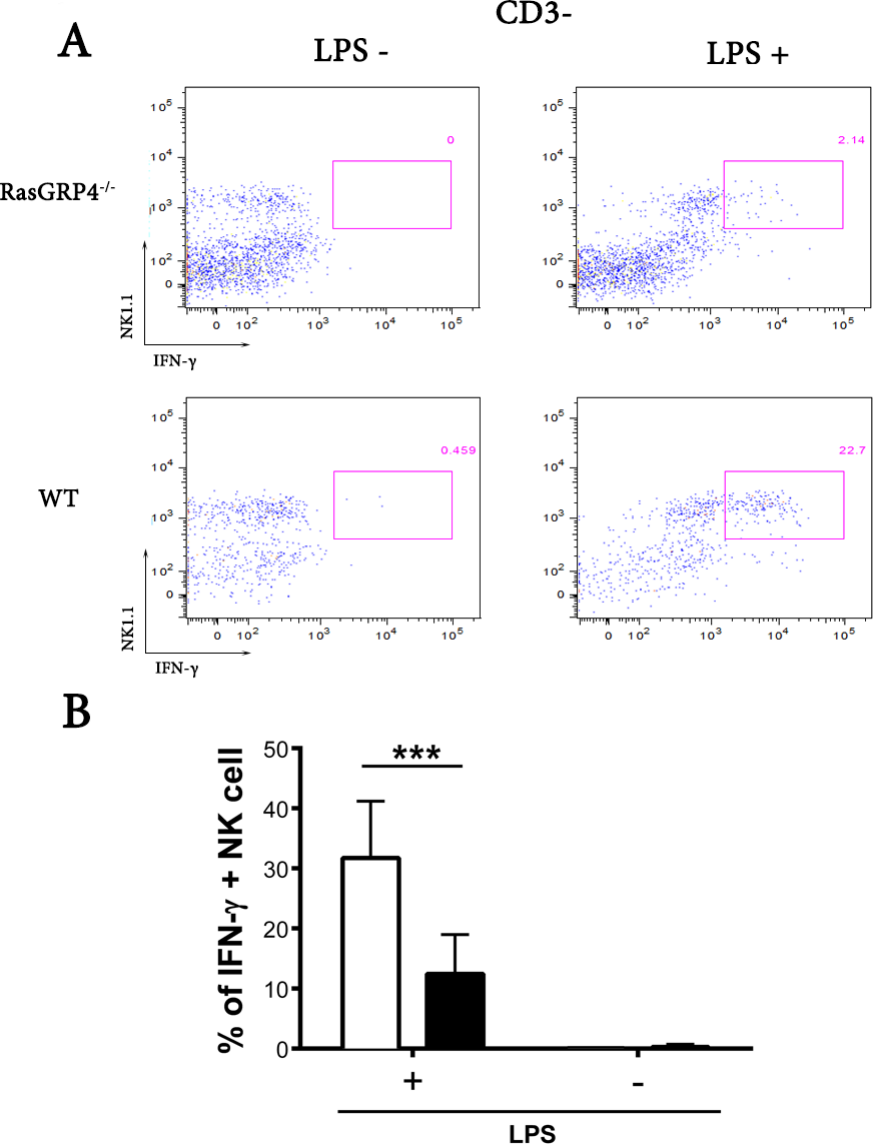
NK cells from RasGRP4-null mice produce significantly less IFN-γ than NK cells from WT mice due to the functional absence of the relevant accessory cell. (A) Splenocytes from RasGRP4-null and WT mice were isolated and incubated 16 h in the absence (left panels) or presence (right panels) of 1 μg/mL of LPS. The numbers of IFN-γ-expressing cells were measured using the intracellular flow cytometry assay. Fewer CD3^-^/NK1.1^+^/IFN-γ^+^ cells were detected in the splenocytes from the RasGRP4-null mice relative to WT mice in this representative experiment. (B) Shown are the mean data ± SD, n = 7 of the percent IFN-γ^+^ NK cells for WT (□) and RasGRP4^-/-^ (■) mice. *** = p< 0.0005.

DCs can induce quiescent NK cells to markedly increase their expression and release of IFN-γ [39]. In order to evaluate the importance of RasGRP4 in IFN-γ production in direct and indirect manners (Fig 2B), we first used flow cytometry and the Miltenyi Biotec mouse IFN-γ secretion assay to identify the major cell type in the spleen that produced this cytokine following LPS challenge. Markedly fewer IFN-γ^+^/CD3^-^/NK1.1^+^ cells were found in the spleens of LPS-treated RasGRP4-null mice relative to WT mice (12.4 ± 2.5% versus 31.7 ± 3.6%, mean ± SD, n = 6, p<0.0005) (Fig 3). Based on these data, we concluded that the NK cells in the spleen of the naïve WT B6 mouse are the primary cells that generate IFN-γ in response to LPS challenge. Nonetheless, our data indicated the presence of an undefined RasGRP4^+^ accessory cell(s) in that same organ.

### Splenic NK cells from WT mice secrete less IFN-γ after co-culture with LPS-responsive RasGRP4-null mBMMCs than WT mBMMCs

mBMMCs express TLRs and are responsive to LPS [21,22]. Although the mechanism was not deduced at the molecular level, Vosskuhl and coworkers reported that LPS-treated mBMMCs induced NK cells to markedly increase their expression of IFN-γ [17]. To determine whether the deficiency of RasGRP4 in mBMMCs affects their ability to regulate IFN-γ production in NK cells by a cross-talk mechanism, RasGRP4-null mBMMCs, WT mBMMCs, and purified NK cells from WT mice were cultured together or alone in the presence of LPS.

**Fig 4:**
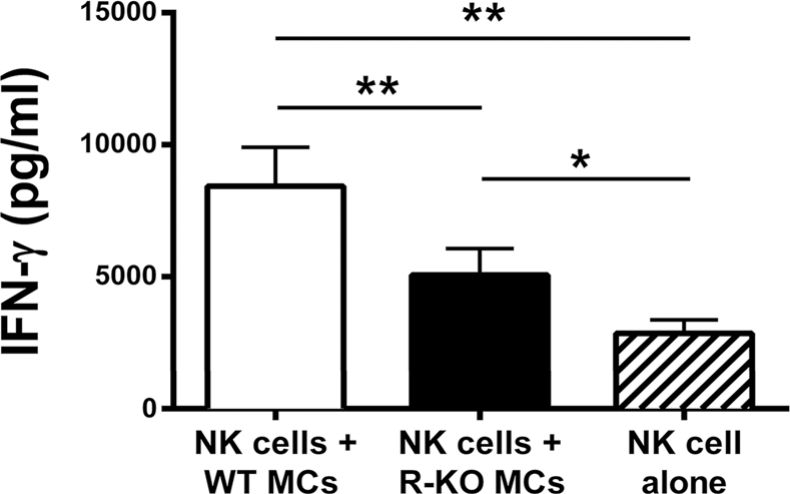
In the presence of LPS, NK cells from WT mice produced significantly less IFN-γ when co-cultured with RasGRP4-null mBMMCs than WT mBMMCs. NK cells were co-cultured in the absence (right hatched bar) or presence of mBMMCs from WT (left open bar) or RasGRP4-null mice (middle closed bar) at a ratio of 1:1 for 24 h in the presence of 1 μg/mL LPS. The levels of IFN-γ in the supernatants were then determined. The depicted data are the mean ± SD, and the results are shown from 3 experiments using different batches of NK cells and mBMMCs. * = p < 0.05, ** = p<0.005.

Prior to these co-culture experiments, we evaluated the expression and release of cytokines and chemokines into the conditioned medium following stimulation of mBMMCs with LPS in the absence of splenic NK cells. WT and RasGRP4-null mBMMCs activated by LPS produced substantial amounts of IL-6 and other cytokines and chemokines, but not IFN-γ. In contrast, WT mouse NK cells stimulated by LPS produced significantly more IFN-γ when co-cultured with WT mBMMCs relative to RasGRP4-null mBMMCs (Fig 4).

To determine whether or not mBMMCs could activate splenocytes depleted of their endogenous CD117^+^ cells (which are primarily DCs as noted below), WT CD117^+^/RasGRP4^+^ mBMMCs and transgenic CD117^+^/RasGRP4^-^ mBMMCs were cultured in the presence or absence of WT mouse splenocytes depleted of their CD117^+^ cells in medium containing or lacking LPS. In these experiments, the CD117^-^ splenocytes (containing NK cells) from WT mice that were co-cultured with WT mBMMCs in the presence of LPS produced 1000 ± 550 pg/mL (mean ± SD, n = 6) and 1750 ± 1100 pg/mL (mean ± SD, n = 6) IFN-γ at the respective 24- and 48-h time points. These IFN-γ values were significantly higher (p< 0.015) than WT mBMMCs cultured in the absence of LPS or RasGRP4-null mBMMCs cultured in the presence of LPS. In regard to the latter, LPS-treated RasGRP4-null mBMMCs co-cultured with CD117^-^ splenocytes from WT mice produced 480 ± 380 pg/mL (mean ± SD, n = 6) and 860 ± 670 pg/mL (mean ± SD, n = 6) at the respective 24- and 48-h time points. Taken together, these data revealed that the ability of LPS-responsive mBMMCs to induce splenic NK cells to optimally increase their expression of IFN-γ was highly dependent on the expression of RasGRP4 in the MCs used in these co-culture cellular-communication experiments.

### The primary CD117^+^ accessory cells in the mouse’s spleen that induce NK cells from WT mice to secrete IFN-γ are DCs, rather than MCs

The mature MCs in B6 mice express varied combinations of mMCP-1 [40,41], mMCP-2 [42], mMCP-4 [43], mMCP-5/mCMA1 [33], and mMCP-6/mTPSB2 [32,44]. Nearly 100,000 ESTs from the spleens of mice have been deposited in GenBank’s EST database. The fact that none of these ESTs originated from the mMCP-1, mMCP-2, mMCP-4, mMCP-5/CMA1, or mMCP-6/TPSB2 genes (see UniGene accession numbers Mm.201549, Mm.4409, Mm.439684, Mm.1252, and Mm.7409, respectively) was strong circumstantial evidence that mature MCs are rarely present in the spleens of naive mice. In support of these data, none of the 53,397 ESTs in the human EST database of the human spleen originated from the corresponding MC-restricted hTryptase-β/TPSB2 and hCMA1 genes (see UniGene accession numbers Hs.592982 and Hs.135626, respectively).

Despite these data, all MCs express CD117 [16] and RasGRP4 [3,5], and LPS-responsive mBMMCs can function as accessory cells for the activation of splenic NK cells in terms of IFN-γ expression (Fig 4). Thus, the possibility had not been ruled out that the primary LPS-responsive accessory cells in the spleen of the naïve WT B6 mouse that induced NK cells to increase their expression of IFN-γ in that organ were a minor population of CD117^+^ MCs.

All examined human [45] and mouse [34] MCs respond to IL-33 due to their surface expression of the cytokine receptor IL1RL1/ST2 [46]. MC also express the high-affinity IgE receptor [47] whose α chain (FcεRIα) [48] is the one which recognizes IgE. In agreement with the EST database of the whole mouse spleen, the levels of the transcripts that encode mMCP-1, mMCP-2, mMCP-4, mMCP-5, mMCP-6, in the CD117^+^ splenocytes isolated from untreated and LPS-treated WT mice (n = 5) were <1% of that of the reference GAPDH transcript (S2 Table). In addition, the levels of the transcripts that encode IL1RL1/ST2 and FcεRIα were below detection (S2 Table). Based on these mRNA data, very few (if any) MCs are present in the spleen of the normal WT B6 mouse. We therefore concluded that the primary cells in the CD117^+^ splenocytes that were needed to induce NK cells to increase their expression of IFN-γ were not MCs.

When the LPS-responsive CD117^+^ cells were purified and characterized from the spleens of WT mice, they had a surface phenotype more indicative of DCs rather than MCs. Consistent with that conclusion, the transcripts that encode many of the cell surface proteins characteristic of different subsets of mouse DCs were quite high in the CD117^+^ splenocytes from WT mice (S4 Table). For example, the migration of mature DCs often is dependent on their expression of the chemokine receptor CCR7 [49]. In that regard, the level of the CCR7 transcript in the CD117^+^ splenocytes purified from naïve WT mice was similar to that of the abundant house-keeping GAPDH transcript. In support of our conclusion that the CD117^+^ cells in the spleen that regulate NK cells were not MCs, mBMMCs do not express CCR7 (see microarray data in Supplemental Table 2 of the Kaieda *et al*. study [34]).

**Fig 5:**
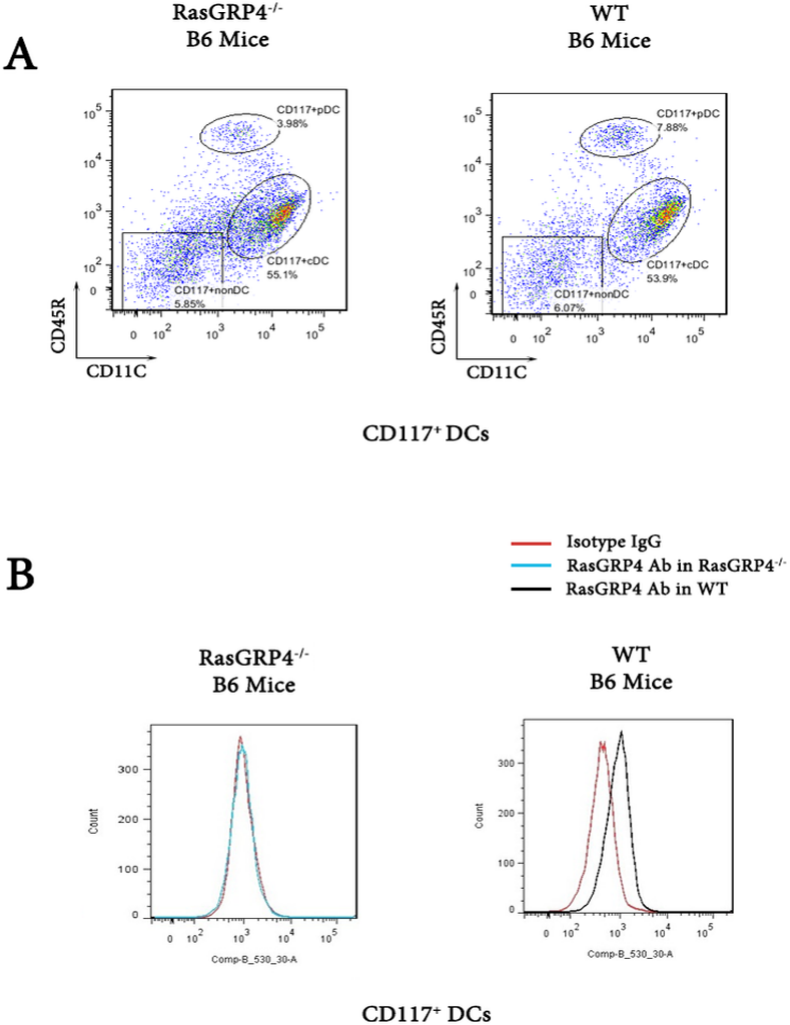
The CD117^+^/CD45R^+^/CD11c^+^ DCs in the spleens of naïve WT B6 mice express RasGRP4. DCs from the spleens of WT B6 and RasGRP4-null mice were stained for their surface expression of numerous proteins, including CD117, CD45R, and CD11c (A), and for their intracellular expression of RasGRP4 (B). An irrelevant immunoglobulin (Isotype IgG) was used as a negative control. Similar data was obtained in a second experiment.

Because the CD117^+^ splenocytes from WT mice contained significant amounts of RasGRP4 mRNA before they were exposed to LPS (S2 Table), we next purified splenic DCs using a cocktail of Abs specifically designed for this purpose. Based on their differential expression of CD45R and CD11c, most of the CD117^+^ DCs in the spleens of WT and RasGRP44-null B6 mice were cDCs rather than pDCs (Fig 5A). Moreover, most of these splenic cDCs contained RasGRP4 protein as assessed immunohistochemically (Fig 5B).

**Fig 6:**
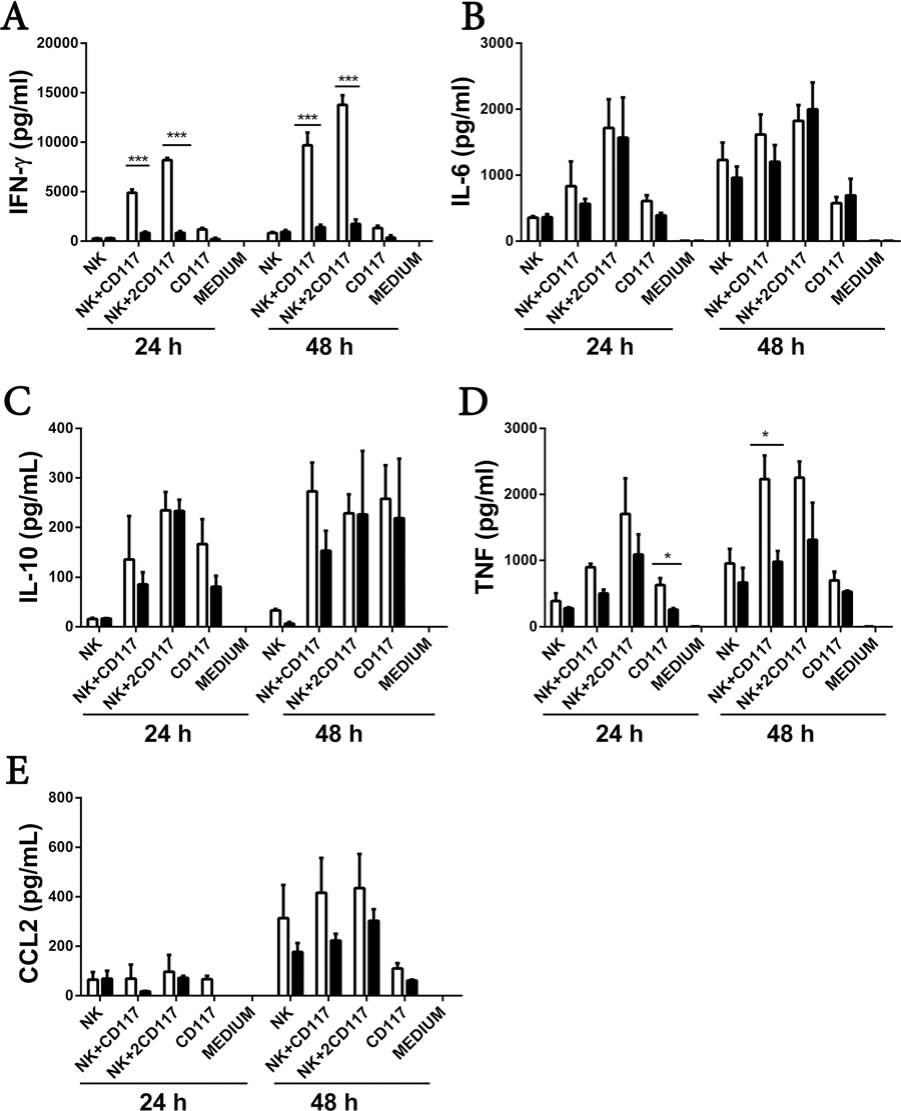
The ability of DC-rich CD117^+^ splenocytes to induce NK cells to increase their expression of IFN-γ in the presence of LPS is dependent on RasGRP4. (A-E) NK cells from WT mice were incubated with DC-rich CD117^+^ splenocytes from WT (□) or RasGRP4- null mice (■) in ratios of 1:1 and 1:2 for 24 and 48 h, respectively, in the presence of 1 μg/mL LPS. For controls, NK cells alone and CD117^+^ cells alone were exposed to LPS. The levels of IFN-γ (A), IL-6 (B), IL-10 (C), TNF/ TNFSF4 (D), and CCL2 (E) in the resulting supernatants were measured. The depicted data are the mean ± SD, and the results are shown from 3 experiments using different batches of NK cells and CD117^+^ splenocytes. * = p < 0.05, ** = p<0.005.

To investigate the regulatory function of RasGRP4 in splenic DCs in terms of their ability to function as accessory cells for the NK cell-mediated production of IFN-γ, we next co-cultured CD117^+^ splenocytes from WT and RasGRP4-null B6 mice with the NK cells from WT mice for 24 or 48 h in the presence of LPS. We then quantitated the levels of numerous cytokines and chemokines in the culture supernatants. As anticipated, the NK cells from WT mice that had been co-cultured with the DC-rich CD117^+^/RasGRP4^+^ splenocytes from WT mice produced ~5 times more IFN-γ at 24 h and ~10 times more at 48 h than DC-rich CD117^+^/RasGRP4^-^ splenocytes (Fig 6A). The former cells also produced more TNF/TNFSF4 at the 48-h time point (mean ± SD, n = 3; p = 0.018) (Fig 6D). In contrast, the levels of IL-6, IL-10, and CCL2 were not significantly different between WT and RasGRP4-null mice (Fig 6B, 6C and 6E).

In the reverse experiment, there was no difference in IFN-γ levels in the supernatants when NK cells isolated from RasGRP4-null mice were co-cultured with the DC-rich CD117^+^/RasGRP4^+^ splenocytes from WT mice (data not shown). The accumulated data revealed that RasGRP4 is needed for the ability of the LPS-stimulated CD117^+^ DCs in the spleen to optimally function as accessory cells for the NK cell-mediated production of IFN-γ in an acute manner, as occurred when MCs encountered NK cells.

### The ability of CD117^+^/RasGRP4^+^ splenocytes to induce NK cells to secrete IFN-γ is not dependent on a TNFSF4 signaling pathway or direct cellular contact

The TNF family member TNFSF4 binds to its receptor TNFRSF4, and it has been reported that the TNFSF4/TNFRSF4 signaling pathway is needed for mBMMCs to optimally induce NK cells to increase their production of IFN-γ in the presence of LPS [17]. While DC-rich CD117^+^/RasGRP4^+^ splenocytes from WT B6 mice increased their levels of TNFSF4 mRNA when exposed 1 h to LPS (S2 and S3 Tables), the same occurred for CD117^+^/RasGRP4^-^ splenocytes. Whether or not LPS-activated CD117^+^ splenocytes also need to physically contact NK cells in order to induce the latter cells to produce high levels of IFN-γ had not been determined. We therefore repeated the preceding co-culture experiment in the presence of an Ab that blocks TNFSF4/TNFRSF4 signaling pathways. There was no significant difference in IFN-γ production by WT mouse NK cells co-cultured with CD117^+^ splenocytes from WT mice at 48 h in the presence or absence of this blocking Ab (data not shown).

Transwell co-culture experiments were next performed to deduce whether direct cellular contact was essential for the splenic CD117^+^ splenocytes to induce NK cells to increase their expression of IFN-γ in the presence of LPS. There was no significant difference in the levels of IFN-γ secreted at either the 24- or 48-h time points between CD117^+^ splenocytes and NK cells from WT mice in these co-culture experiments regardless of whether or not the two cell types were allowed to physically contact one another (data not shown). The accumulated data revealed that the LPS-dependent activation of the *in vivo*-differentiated CD117^+^/RasGRP4^+^ DCs in the spleen results in the release of an unknown soluble factor that induces NK cells to markedly increase their expression of IFN-γ.

### NK cells from the peripheral blood and spleens of WT mice, but not RasGRP4-null mice, secrete IFN-γ after *in vivo* stimulation with LPS

**Fig 7:**
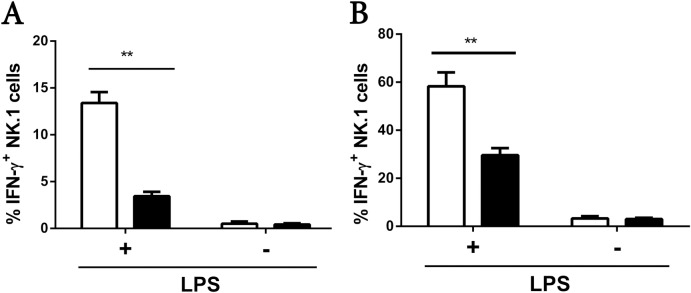
RasGRP4 is needed for the ability of the NK cells in the peripheral blood and spleens of LPS-treated animals to express high levels of IFN-γ. (A-B) WT (□) and RasGRP4^-/-^ (■) B6 mice were euthanized 2.5 h after they were given LPS. The percentages of IFN-γ^+^ NK cells in the each animal’s peripheral blood leukocytes (A) and spleen (B) were then determined. The graphs represent mean ± SD, n = 3. ** = p < 0.005.

We next assessed the *in vivo* relevance of our *in vitro* data. There was no difference in the total numbers of peripheral blood and splenic NK1.1^+^ cells between WT and RasGRP4-null mice given LPS intravenously when expressed as a percentage of total white cells and splenocytes, respectively (data not shown). In contrast, there was a significant difference in the percentage of peripheral blood and splenic NK1.1 cells that expressed IFN-γ in WT mice compared to RasGRP4-null mice following LPS injection (Fig 7A and 7B).

## Discussion

Although the expression of RasGRP4 is more restricted than RasGRP1, RasGRP-2, and RasGRP3, this intracellular signaling protein is present in every examined mouse, rat, and human MC [3]. We now show that RasGRP4 is necessary for WT mBMMCs to induce NK cells to optimally increase their expression of IFN-γ in the presence of LPS. The unexpected finding that the *in vivo*-differentiated CD117^+^ cDCs in the spleens of WT mice expressed RasGRP4 raised the possibility that this GTPase might also dictate DC-mediated activation of NK cells in terms of their expression of the cytokine IFN-γ. In fact, when we phenotyped the CD117^+^ population in the mouse’s spleen, we found that DCs, rather than MCs, were the main population of RasGRP4^+^/CD117^+^ cells in that organ responsible for the enhanced IFN-γ secretion by NK cells. The microarray mRNA data supported this conclusion.

MCs and DCs can regulate NK cells directly via cell-to-cell contact or indirectly by the release of NK cell-responsive soluble factors [50]. It has been shown that some cytokines (e.g., IL-12, IL-15, IL-18, and IL-10) are regulators of LPS-induced IFN-γ production [38]. However, we did not observe any significant difference in the levels of any of these mediators when WT and RasGRP4-null CD117^+^ DCs were exposed to LPS.

Prostaglandin-endoperoxide synthase 1 participates in the biosynthesis of prostaglandins. The level of transcript that encodes this enzyme markedly decreased in the DC-rich CD117^+^ splenocytes from WT and RasGRP4-null mice when these cells encountered LPS for 24 h (see probe set 17370309 data in S2 Table). Nevertheless, because RasGRP4 regulates prostaglandin D_2_ expression in transformed rat and human MC lines [6] and because LPS induces certain populations of human DCs to increase their expression of prostaglandin E_2_ [51], the possibility has not been ruled out that the primary MC- and DC-derived soluble mediator that induced NK cells to express IFN-γ was a prostaglandin or some other biologically active lipid.

In terms of the relevance of our new data to inflammatory diseases, it is now possible that the findings that RasGRP4 participates in experimental arthritis and colitis [9] could be a consequence of dysregulation of DCs rather than MCs or synovial fibroblasts. RasGRP4 is an oncogenic protein. Transfection of fibroblasts with RasGRP4 expression constructs results in their loss of contact inhibition [3,5,52], and the synovial fibroblasts in a subset of arthritis patients aberrantly express RasGRP4 [53]. Moreover, many of the RasGRP4 ESTs in the human database originated from clear cell tumors of the kidney, germ cell tumors, and different leukemias (see GenBank UniGene Hs.130434). Despite the adverse roles of RasGRP4 in these diseases, we now show that RasGRP4 participates in acute LPS-TLR-signaling pathways in MCs and DCs, and that this intracellular protein participates in innate immunity by controlling the expression of undefined soluble factors released from the latter cells that are essential for NK cells to produce optimal amounts of IFN-γ.

Finally, the control mechanisms that downregulate MC- and/or DC-NK cell crosstalk have not been identified. In that regard, the levels of the RasGRP4 transcript in the DC-rich CD117^+^ splenocytes from WT B6 mice markedly decreased 12–48 h after these cells encountered LPS (S2 Table). These findings raise the possibility that the LPS-mediated downregulation of RasGRP4 expression in DCs is a novel way DC-NK cell cross-talk is regulated to prevent the development of life-threatening hyperactive immune responses to pathogens.

## Supporting Information

S1 Table Conjugated Abs and isotype controls used for flow cytometry. (DOCX)

S2 Table Microarray data of DC-rich CD117^+^ splenocytes from RasGRP4-null and WT B6 mice exposed to LPS. (XLSX)

S3 Table Transcripts preferentially increased in the CD117^+^ splenocytes from WT and RasGRP4-null B6 mice cultured in the presence of 1 μg/ml LPS for 24 h. (XLSX)

S4 Table Typical DC transcripts present in the CD117^+^ splenocytes of naïve RasGRP4-null and WT B6 mice. (XLSX)
