# Supplementary material for: CD117+ Dendritic and Mast Cells Are Dependent on RasGRP4 to Function as Accessory Cells for Optimal Natural Killer Cell-Mediated Responses to Lipopolysaccharide
Source: PLoS One. 2016 Mar 16;11(3):e0151638. doi: 10.1371/journal.pone.0151638 (PMC4794117; doi:10.1371/journal.pone.0151638)
Supplement: S1 Table — (DOCX) [file pone.0151638.s001.docx]

**S1 Table. Conjugated Abs and their isotype controls used for flow cytometry.**

| **Surface protein** | **Conjugate** | **Clone** | **Species** |
| --- | --- | --- | --- |
| CD1d | FITC | 1B1 | Rat (LEW) IgG2b, κ |
| CD3e | V500 | 500A2 | Syrian Hamster IgG2, κ |
| CD4 | V500 | RM4-5 | Rat (DA) IgG2a, κ |
| CD8a | Alexa Fluor 488 | 53-6.7 | Rat (LOU) IgG2a, κ |
| CD11b | APC-Cy7 | M1/70 | Rat (DA) IgG2b, κ |
| CD11b | FITC | M1/70 | Rat (DA) IgG2b, κ |
| CD11c | PerCP-Cy5.5 | HL3 | Armenian Hamster IgG1, λ2 |
| CD11c | PE | N418 | Armenian Hamster IgG |
| CD14 | APC | rmC5-3 | Rat (LOU) IgG1, κ |
| CD21/35 | APC | 7G6 | Rat (SD) IgG2b, κ |
| CD24 | Brilliant Violet 421 | M1/69 | Rat (DA) IgG2b, κ |
| CD25 | APC | 3C7 | Rat (LEW) IgG2b, κ |
| CD34 | FITC | RAM34 | Rat IgG2a, κ |
| CD38 | PE | 90/CD38 | Rat IgG2a, κ |
| CD45R/B220 | Pacific Blue | RA3-6B2 | Rat IgG2a, κ |
| CD45R/B220 | PE-Cy7 | RA3-6B2 | Rat IgG2a, κ |
| CD45R/B220 | Brilliant Violet 570 | RA3-6B2 | Rat IgG2a, κ |
| CD117 | APC | 2B8 | Rat (WI) IgG2b, κ |
| CD117 | PE-Cy7 | 2B8 | Rat (WI) IgG2b, κ |
| CD117 | Biotin | 2B8 | Rat (WI) IgG2b, κ |
| CD172a (SIRPα) | PE | P84 | Rat IgG1, κ |
| CD197 (CCR7) | Alexa Fluor 488 | 4B12 | Rat IgG2a, κ |
| CD205 | PE-Cy7 | NLDC-145 | Rat IgG2a, κ |
| CD252 (OX40L) | PE | RM134L | Rat IgG2b, κ |
| IFNγ | APC | AN18.17.24 | Rat IgG1 |
| Ly-6A/E (Sca-1) | PE-Cy7 | D7 | Rat (LEW) IgG2a, κ |
| Ly-6B | PE | 7/4 | Mouse IgG2a |
| Ly-6C | APC-Cy7 | AL-21 | Rat IgM, κ |
| Ly-6G | FITC | 1A8 | Rat (LEW) IgG2a, κ |
| Ly-6G and Ly-6C (Gr-1) | V450 | RB6-8C5 | Rat IgG2b, κ |
| NK1.1 | FITC | PK136 | Mouse IgG2a, κ |
| Isotype control | V500 | B81-3 | Armenian Hamster IgG2 , ƙ |
| Isotype control | PE | R3-34 | Rat IgG1, κ |
| Isotype control | PerCP-Cy5.5 | G235-2356 | Armenian Hamster IgG1, λ2 |
| Isotype control | APC | A95-1 | Rat IgG2b, κ |
| Isotype control | PE-Cy7 | R35-95 | Rat IgG2a, κ |
| Isotype control | FITC | G155-178 | Mouse IgG2a, κ |
| Isotype control | FITC | A95-1 | Rat IgG2a, κ |
